# Delineation of Concentration Ranges and Longitudinal Changes of Human Plasma Protein Variants

**DOI:** 10.1371/journal.pone.0100713

**Published:** 2014-06-23

**Authors:** Olgica Trenchevska, David A. Phillips, Randall W. Nelson, Dobrin Nedelkov

**Affiliations:** 1 Molecular Biomarkers Laboratory at the Biodesign Institute, Arizona State University, Tempe, Arizona, United States of America; 2 Intrinsic Bioprobes, Tempe, Arizona, United States of America; 3 Institute for Population Proteomics, Tempe, Arizona, United States of America; California Institute of Technology, United States of America

## Abstract

Human protein diversity arises as a result of alternative splicing, single nucleotide polymorphisms (SNPs) and posttranslational modifications. Because of these processes, each protein can exists as multiple variants *in vivo*. Tailored strategies are needed to study these protein variants and understand their role in health and disease. In this work we utilized quantitative mass spectrometric immunoassays to determine the protein variants concentration of beta-2-microglobulin, cystatin C, retinol binding protein, and transthyretin, in a population of 500 healthy individuals. Additionally, we determined the longitudinal concentration changes for the protein variants from four individuals over a 6 month period. Along with the native forms of the four proteins, 13 posttranslationally modified variants and 7 SNP-derived variants were detected and their concentration determined. Correlations of the variants concentration with geographical origin, gender, and age of the individuals were also examined. This work represents an important step toward building a catalog of protein variants concentrations and examining their longitudinal changes.

## Introduction

Human protein diversity arises as a result of three main processes: i) Alternative splicing, which can produce multiple protein products from a single gene [1]; ii) Non-synonymous single nucleotide polymorphisms (SNPs) [2] that can produce single amino acid variations [3]; and iii) Posttranslational modifications (PTMs) which can add or remove chemical moieties on either an amino acid or the protein N- or C-terminus [4]. Because of these processes, each protein can exist as multiple variants *in vivo*, thus making the human proteome far more complex than the human genome and its ∼25,000 human genes. Therefore, tailored strategies are needed to study the protein diversity and understand its role in health and disease.

Protein variants were initially studied via gel electrophoresis. One-dimensional gel isoelectric focusing was the method of choice, contributing to the discovery of variants for proteins such as hemoglobin [5], alpha-antitrypsin [6], amylase [7], and prealbumin (transthyretin) [8]. Subsequently, two-dimensional gel electrophoresis provided increased resolving power for simultaneous analysis of hundreds of proteins and their variants, especially when coupled to mass spectrometric (MS) identification of the protein spots from the gel [9]. Two-dimensional difference gel electrophoresis (DIGE) has been used in delineating protein variations across populations, as shown in the study of longitudinal and individual variation of 78 proteins from eleven healthy subjects [10], the normal variability of the human plasma proteome from 60 healthy subjects [11], and age-related differences in 100 plasma proteins from 42 individuals [12]. Because of dynamic range, reproducibility and throughput limitations, 2D gel electrophoresis was surpassed by bottom-up and top-down mass spectrometry approaches for decoding protein modifications [13]–[15]. In bottom-up approaches, proteins are digested with trypsin, and selected reaction monitoring (SRM) or multiple reaction monitoring (MRM) mass spectrometry is used to detect peptides (straight from the human plasma, or immuno-enriched) that contain the protein modifications. Osteopontin splice variants were identified and quantified using MRM-MS [16]; novel proteoforms of prostate specific antigen were also identified in clinical samples via MRM-MS [17]. In a larger population study, the concentration of three selected single amino acid polymorphism peptides, representing the Complement Component C7, Complement Factor H, and Complement Component C5 proteins, were measured by SRM-MS from 290 individuals [18]. In another population study, serum peptide variations were studied in 500 healthy individuals using a regular MS analysis (without trypsin digestion), but the peptides detected were exopeptidase products derived from relatively abundant serum proteins [19].

Top-down MS approaches provide more accurate and complete results for protein variants identification because there is no prerequisite for *a priori* knowledge of the protein modification in order to select the appropriate modification-specific peptide. But analyzing and quantifying intact proteins and their modifications with mass spectrometry can be challenging [20]. Our group has devised a simple method termed mass spectrometric immunoassay [21] that combines a one-step affinity protein isolation with MS analysis that is ideally suited for high-throughput analysis of human plasma proteins and their variants [22]. Antibodies are surface-immobilized in small, porous microcolumns that are fitted at the entrance of a pipettor tip. Samples are passed through the pipette tip repetitively until enough protein is bound to the antibody. Following washing with buffer and/or other mild solutions to remove non-specifically bound sample components, the proteins are eluted with a small volume of matrix solution and deposited directly onto a MALDI target for ensuing MS analysis.

We’ve applied this approach to investigate the diversity of 25 human plasma proteins from a cohort of 96 healthy individuals, resulting in the detection of 76 structural variants, each occurring with different frequencies [23]. Subsequently, we’ve screened 1,000 individuals from four geographical regions in the United States, and determined the variants and their frequencies for five proteins - beta-2-microglobulin, cystatin C, retinol binding protein, transferrin, and transthyretin [24]. The qualitative data from those studies provided a first glimpse into the extent of protein structural diversity in the general population.

Recently we have developed and validated fully quantitative mass spectrometric immunoassays for 4 of those clinically relevant proteins - beta-2-microglobulin [25], cystatin C [26], retinol binding protein [27] and transthyretin [28]. Beta-2-microglobulin is used in the diagnosis of active rheumatoid arthritis and kidney disease [29], [30], and a structural variant of b2m has been associated with autoimmune disease and small-cell lung cancer [31], [32]. Cystatin C is a serine proteinase inhibitor with implications in renal failure [33], [34]. Retinol binding protein has also been implicated in renal disease [35], with the increased presence of its truncated variants suggested as indication of renal failure [36]. Transthyretin has been associated with systemic amyloidoses and familial amyloidotic polyneuropathy [37], [38], primarily as a result of its more than 80 SNPs [39]. The mass spectrometric immunoassays for these 4 proteins were designed to detect and quantify individual protein variants. In this work we’ve applied these four quantitative assays to determine the concentration of all variants we can detect in 500 healthy individuals. Additionally, we determined the longitudinal concentration changes over a 6 month period for those variants in four individuals.

## Materials and Methods

### Reagents

Polyclonal rabbit anti-human antibodies to beta-2-microglobulin (b2m, Cat. No A0072, 5.7 g/L), cystatin C (cysC, A0451, 17 g/L), retinol binding protein (RBP, A0040, 4.1 g/L), and transthyretin (TTR, A0002, 3.9 g/L) were obtained from DAKO (Carpinteria, CA, USA). Rabbit anti-human polyclonal antibody to beta-lactoglobulin (BL) was obtained from GeneTex (Irvine, CA, GTX77272, 1 mg/mL). Recombinant human beta-2-microglobulin (b2m) was purchased from Cell Sciences (Canton, MA, CSI19620). Recombinant human cystatin C (cysC) was purchased from HyTest (Turku, Finland, 8CY5). Purified human retinol binding protein (RBP) was purchased from Sigma (St. Louis, MO, R9388). Beta-lactoglobulin from bovine milk (L8005), 1,1′ Carbonyldiimidazole (115533), TWEEN 20 (P7949), TRIS (T-6128), and α-cyano -4-hydroxycinnamic acid (476870) were obtained from Sigma-Aldrich (St. Lous, MO). Sinapinic acid (3-(4-hydroxy-3,5-dimethoxyphenyl)prop-2-enoic acid)was purchased from Fluka (Cat. No. 85429). Affinity pipettes fitted with porous microcolumns were obtained from Intrinsic Bioprobes (Tempe, AZ, IBI-CMD-R96). Phosphate buffered saline (PBS) was obtained from Thermo Scientific (Rockford, IL, 28374). HBS-EP buffer (0.01 M HEPES pH 7.4; 0.15 M NaCl, 3 mM EDTA, 0.005% v/v Surfactant P20) was obtained from GE Healthcare (Piscataway, NJ). Sterile water (AB02120), acetone (AB00636), MES (AB01235), acetonitrile (AB00120) and trifluoracetic acid (AB02010) were purchased from American Bionalytical (Natick, MA). 1-Methyl 2-pyrrolidone was obtained from EMD Chemicals (Gibbstown, NJ, MX1932-5). *N*-octylglucoside was obtained from Roche Applied Science (Indianapolis, IN, 10634425001). Sequencing grade modified trypsin was obtained from Promega (Madison, WI, V511). Sequencing grade endoproteinase Arg-C was obtained from Roche Applied Science (Penzberg, Germany, 11370529001).

### Human plasma samples

Five hundred sodium heparin human plasma samples were obtained from ProMedDX (Norton, MA, USA), and were designated as normal based on their non-reactivity for common blood infectious agents and the donor information itself. The samples originated from two states: Tennessee (TN, 247 samples, 210 male, 37 female, median age 37), and Texas (TX, 253 samples, 201 males, 52 females, median age 36). The samples were received labeled only with a barcode and supplied with an accompanying specification sheet containing information about the gender and age. For the longitudinal study, human capillary blood was obtained once a week (fasting morning blood draw), for twenty seven weeks, from 4 individuals (three males, one female, median age 36). Each individual had signed an Informed Consent Form. This study was approved by Intrinsic Bioprobes' Institutional Review Board (IRB No. 00001399). Seventy-five microliters of blood were drawn under sterile conditions from a lancet-punctured finger with heparinized microcolumn (Drummond Scientific Co., Broomal, PA), and centrifuged for 60 s at 2,500g to pellet red blood cells. The supernatant plasma was stored at −80°C until used.

### Assay execution and data analysis

The antibody-derivatized affinity pipettes, analytical solutions of the proteins for the standard curves, internal reference standard (beta lactoglobulin), plasma samples dilutions, and analytical samples solutions, were prepared as described previously for each of the four assays [25]–[28]. The antibody-derivatized affinity pipettes were mounted onto the head of a Multimek 96 automated 96-channel pipettor (Beckman Coulter, Brea, CA) and initially rinsed with assay buffer (PBS or HBS-EP, 10 aspirations and dispense cycles, 100 μL volumes each). Next, the pipettes were immersed into a microplate containing the samples and 100 aspirations and dispense cycles (200 for RBP) were performed (100 µL volumes each) allowing for affinity capture of the targeted proteins. The pipettes were then rinsed with assay buffer (100 cycles), and twice with water (10 cycles each). For elution of the captured proteins, 6 µL aliquots of MALDI matrix (25 g/L α-cyano-4-hydroxycinnamic acid, or 13.3 g/L sinapinic acid, in aqueous solution containing 33% (v/v) acetonitrile, and 0.4% (v/v) trifluoroacetic acid) were aspired into the affinity pipettes, and after a 10 second delay (to allow for the dissociation of the protein from the capturing antibody), the eluates from all 96 affinity pipettes containing the targeted proteins were dispensed directly onto a 96-well formatted MALDI target. Following air-drying and visual inspection of the sample spots, linear mass spectra were acquired on an *Autoflex* II MALDI-TOF mass spectrometer (Bruker, Billerica, MA), with a delayed extraction mode using a 1.7 kV draw out pulse, 200 ns delay, and a full accelerating potential of 20 kV. Five mass spectra were acquired from each sample spot (to gain representative sample data), each spectrum consisting of three-hundred laser shots. The mass spectra were processed (baseline subtracted and smoothed) with Flex Analysis software (Bruker).

We measured peak heights and used them for quantification. The peak heights for the protein signals and the internal reference standard (beta lactoglobulin - BL) were determined from each spectrum and entered into an Excel spreadsheet. The protein/BL peak heights ratios were calculated, and the average protein/BL ratio for each sample was determined from the five mass spectra. A standard curve was generated for each protein by plotting the average standard protein/BL ratios against the concentration of the protein standards, and the data was fitted with a linear trendline using Sigma Plot (Systat Software, San Jose, CA). The standard curves were then utilized to determine the concentration of the four proteins and their variants in the analytical samples. We first determined the protein/BL peak heights ratios for each variant, summed up the ratios of all variants for an individual protein, determined the total individual protein concentration using the corresponding standard curve, and then determined the concentration of each variant based on its percentage of the total protein concentration. This approach worked especially well for those variants whose variant/BL peak ratios were below those of the standard curve. Each sample was analyzed in duplicates, at two various dilutions, and the final concentrations were determined as an average of the two measurements (if the two measurements were significantly different, the assays were repeated). For identification of the TTR variants, enzymatic digestion with trypsin and endoproteinase Arg-C was performed *in situ* directly on the TTR eluates deposited onto the MALDI target after the mass spectrometric immunoassay of the human serum samples, as described in the TTR assay development paper [28]. Reflectron mass spectra were acquired on an *Autoflex* II *TOF/TOF* MALDI-TOF mass spectrometer (Bruker, Billerica, MA), with a delayed extraction mode, using a 2.1 kV draw out pulse, 1100 ns delay, an ion mirror voltage of 20 kV, and a full accelerating potential of 19 kV.

## Results and Discussion

**Figure 1:**
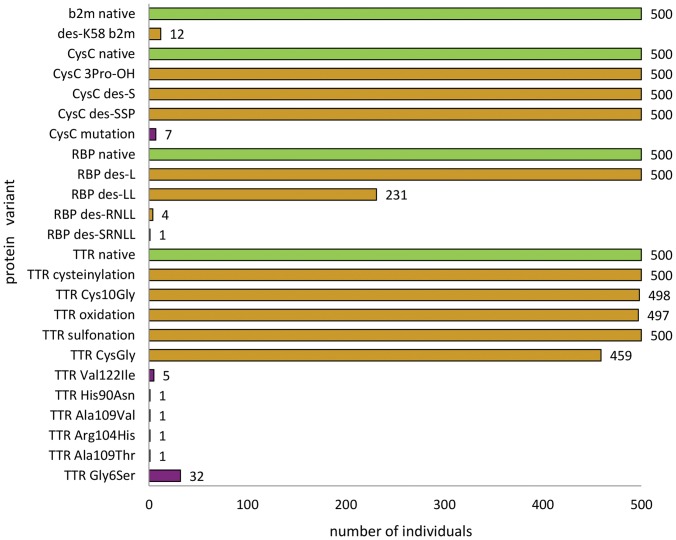
Proteins and their variants detected among the 500 individuals. Green: native proteins; Yellow: posttranslational modifications; Purple: SNP-derived variants.

Summarized in **Fig. 1** and **Table 1** are all the protein variants detected with the four assays, and their frequency among the 500 individuals. Along with the native forms of the four proteins, 13 posttranslationally modified variants and 7 SNP-derived variants were detected, for a total of 24 distinct protein species. For b2m only one posttranslationally modified form was detected – generated by the cleavage at position 58 in the amino acid sequence and removal of Lys58 (des-K58) [40]. This variant was detected in 12 individuals (2.4% of the samples), which is in agreement with our previous observations of 2–6% occurrence in the normal population [23], [24]. Cystatin C was detected in its native form and as 3 posttranslationally modified variants in all 500 samples: one variant contained hydroxyproline at position 3 (3Pro-OH) [41], and two others contained truncated sequences – the first one missing its N-terminal serine (des-S), and the second one missing three N-terminal residues (des-SSP). An SNP-derived variant was also detected for cysC, as evidenced by a shift of -30Da in the mass spectra. This SNP was observed only in male subjects, as in our previous work, and with similar frequencies [23], [24]. Retinol binding protein was observed as 4 posttranslationally modified variants, derived by C-terminal truncation of Leu (des-L), Leu-Leu (des-LL), Arg-Asn-Leu-Leu (des-RNLL), and Ser-Glu-Arg-Asn-Leu-Leu (des-SERNLL). The frequencies of these variants in the 500 samples matched those observed in our previous studies [23], [24]. Finally, TTR exhibited the most diversity. Five posttranslationally modified variants were observed in most samples, resulting from the reactive Cys10 residue that yielded variants with cysteinylation, cysteine10glycine transformation (Cys10Gly), oxidation, sulfonation, and cysteinyl-glycine (CysGly). In addition, six SNP-derived variants were observed for TTR, and confirmed via enzymatic *in situ* digestion. All of the SNPs have been described previously [39]. Of note, the Gly6Ser variant was observed in 32 samples (6.4%), which is in a very good agreement with the previous results that yielded frequencies of 7% [23] and 5.6% [24]. Representative mass spectra depicting the protein variants are shown in **Fig. S1**.

**Table 1:** Molecular weights of the observed proteins and their variants.

Protein	Molecular Weight
Beta-2-microglobulin	11,732.0
des-K58 beta-2-microglobulin	11,618.0
Cystatin C	13,343.1
Cystatin C 3Pro-OH	13,359.0
Cystatin C des-S	13,256.0
Cystatin C des-SSP	13,071.8
Cystatin C mutation	13,315.2
Retinol binding protein	21,065.5
Retinol binding protein des-L	20,952.4
Retinol binding protein des-LL	20,839.2
Retinol binding protein des-RNLL	20,568.9
Retinol binding protein des-SERNLL	20,352.7
Transthyretin	13,761.4
Transthyretin cysteinylation	13,880.4
Transthyretin Cys10Gly	13,715.4
Transthyretin oxidation	13,793.4
Transthyretin sulfonation	13,841.4
Transthyretin CysGly	13,937.4
Transthyretin Val122Ile	13,775.4
Transthyretin His90Asn	13,738.4
Transthyretin Ala109Val	13,789.4
Transthyretin Arg104His	13,742.4
Transthyretin Ala109Thr	13,791.4
Transthyretin Gly6Ser	13,791.4

**Figure 2:**
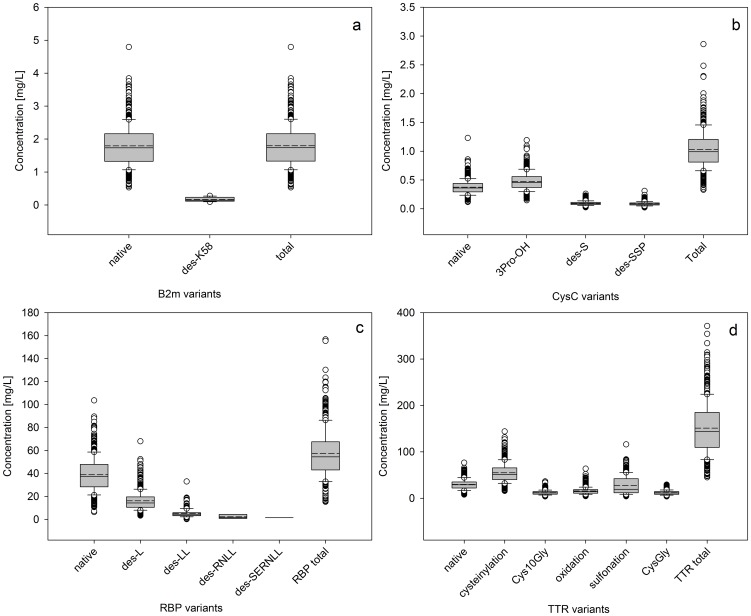
Individual concentrations of (a) b2m, (b) CysC, (c) RBP, and (d) TTR, and their variants among the 500 individuals. Box - 25-75^th^ percentile; Solid line – median concentration; Short dash line – mean concentration; Error bars – 10^th^ and 90^th^ percentile; Symbols – outlying points.

Using standard curves (examples of which are shown in **Fig. S2**) we determined the individual concentrations of each native protein and its variants in the 500 individuals. The data are presented in **Fig. 2** as box plots with the statistical measurements of median, mean, 25^th^ and 75^th^ percentile, 90^th^ and 10^th^ percentile, and outlying points. For b2m and RBP, the native protein was present at the highest concentration, and was by far the most dominant form of the protein. For cysC and TTR, however, the 3Pro-OH and the cysteinylated variants, respectively, were the most abundant species; the native forms of these two proteins were only a small fraction of the total proteins present *in vivo.* Considering the total protein concentrations (obtained by summing up the concentration of the native form and the variants), the values obtained were well within the reference range for each protein in a healthy population: ∼2 mg/L for b2m [30], ∼0.85 mg/L for cysC [42], ∼40 mg/L for RBP [43], and 150–250 mg/L for TTR [44], all determined by traditional enzymatic immunoassays.

**Figure 3:**
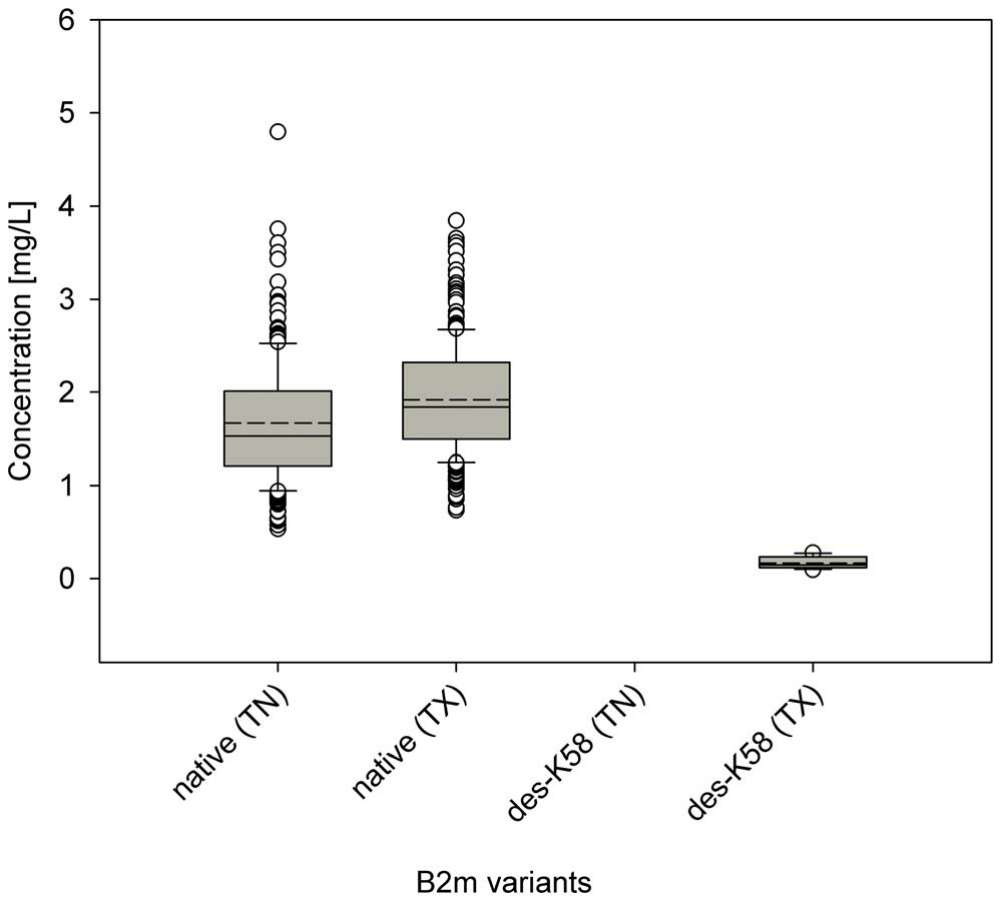
Concentrations of b2m and its des-K58 variant among the 500 individuals as a function of the state of origin (TN and TX).

**Figure 4:**
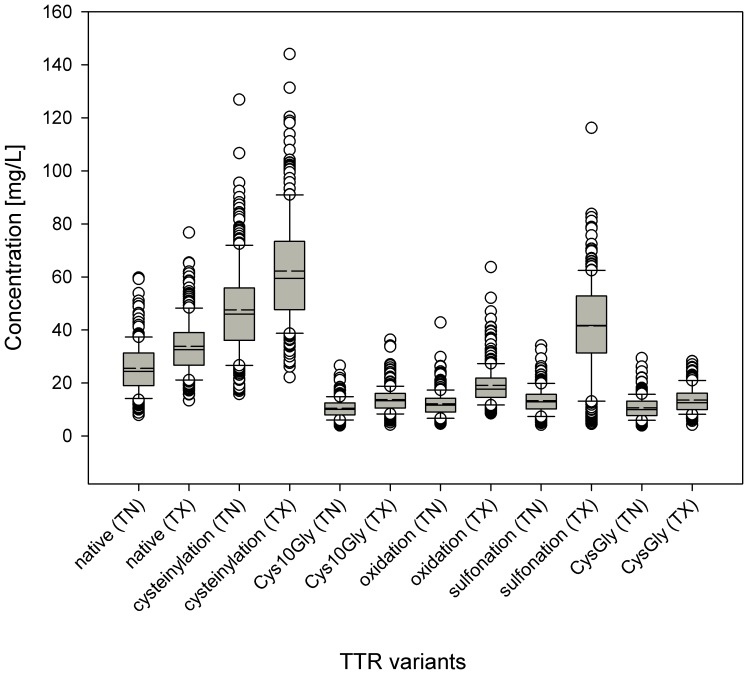
Concentrations of TTR and its variants among the 500 individuals as a function of the state of origin (TN and TX).

The concentration data were further parsed in regards to the geographical origin (state), gender, and age of the individuals. The 500 human plasma samples were obtained from 411 male (M) and 89 female (F) individuals; the median and mean ages of all the individuals were 36 and 37, correspondingly; 247 samples were collected at hospitals in Tennessee (TN), and 253 in Texas (TX). Several interesting correlations were derived for these attributes. For b2m, the des-K58 variant was present in a small number of samples (twelve), at very low concentration, and only in individuals from TX (**Fig. 3**). Even though the TX samples had on average higher native b2m concentration, the presence of the des-K58 b2m variant was not related to the total expression of b2m, as the des-K58 variant appeared in individuals with total b2m concentration ranging from 1.28 to 2.98 mg/L. All cysC variants were present at slightly elevated concentrations in the samples from TX, and the same trend was observed for the native form of RBP (**Fig. S3**). This elevated protein concentrations in the TX samples was most prominent for TTR where the concentration of the sulfonated variant was much higher than that seen in the TN samples (**Fig. 4**). It seems very unlikely that the individuals from Texas have truly elevated forms of these proteins. Because both the TN and TX samples were collected in the same time period (TN: January-February 2009; TX: March-April 2009), a more likely explanation is that the samples were handled somewhat differently – either the collection tubes were from a different manufacturer, or the plasma samples were stored differently post-collection before being placed at −80°C for long term storage. Whatever the cause, the evaluation of the data points out that standardized sample collection and storage protocols are important, because today's MS-based techniques can easily reveal the slightest differences due to non-conformity and deviations from the standard sample-handling protocols.

**Figure 5:**
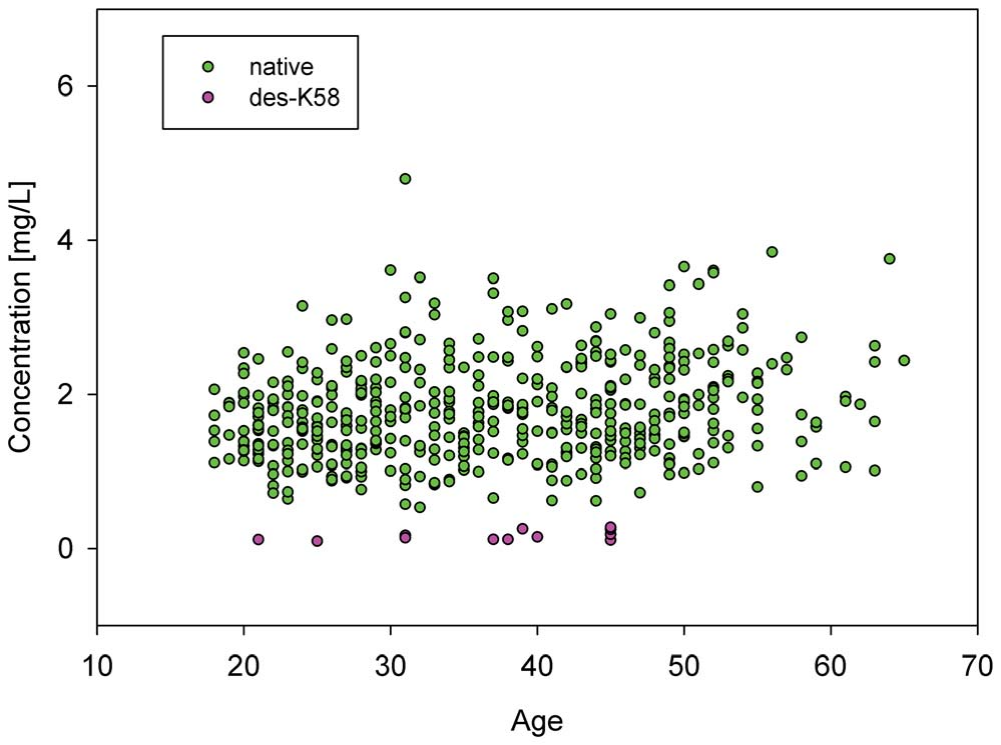
Concentrations of b2m and its des-K58 variant among the 500 individuals as a function of age.

In regards to the gender of the individuals (**Fig. S4**), slightly elevated levels in native cysC and its 3Pro-OH variant were seen in males compared to females, with the same trend observed for native and des-L RBP. As for the age, shown in **Fig. 5** is the concentration distribution of b2m and its des-K58 variant as a function of age. There isn’t any significant age correlation, and similar observations were made for the other three proteins and their variants in regards to the age of the individuals (**Fig. S5**).

**Figure 6:**
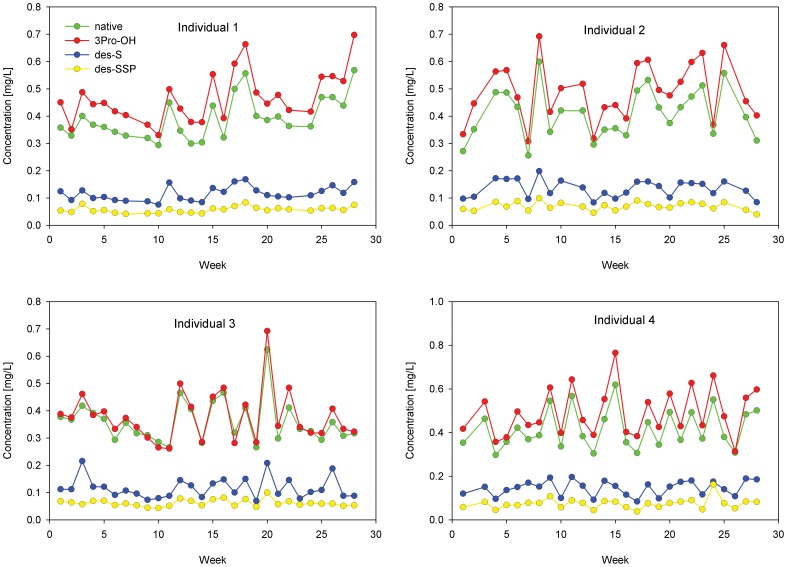
CysC variants longitudinal concentration profiles for the four individuals over a 27-week period.

For the longitudinal study of the protein variants, we enrolled 4 individuals and collected heparinized plasma samples once a week, for twenty seven weeks, from a fasting morning blood draw. We analyzed the samples (28 time points per individual) with the four assays and determined the concentration of all protein variants. Presented in **Fig. 6** are the cysC variants longitudinal concentration profiles for the four individuals. The concentrations of all cysC forms oscillate as much as two-fold, although the values are well within the normal concentration range for totals cysC (as measured by ELISAs). The profiles for the other 3 proteins are shown in **Fig. S6**. Notably, none of the individuals exhibited the des-K58 b2m variant, nor any of the SNP-derived variants of TTR.

To the best of our knowledge, this is the first detailed study of protein variants, their concentration range, and longitudinal change among a larger cohort from a healthy population. This study was made possible by the high-throughput method of mass spectrometric immunoassay. In practice, mass spectrometric immunoassays are no more complicated than standard enzymatic immunoassays (i.e. ELISAs): the first step (immunoaffinity capture) is identical; the difference is in the secondary, detection step. The use of mass spectrometry enables unambiguous protein variants detection, which enzymatic immunoassays cannot do. However, the key to a successful implementation of MS for larger scale population studies of proteins and their variants is the sample-MS interface, that is, the presentation of the proteins for MS detection. Several methodologies exist in that regard: in one approach, the entire human sample is fractionated via LC/LC separations before introduction into the mass spectrometer [45]; in another, the entire sample is proteolyzed and 1–2 surrogate peptides are detected with MS for protein quantification [46]; yet another approach utilizes immuno-retrieval of intact proteins, only to subsequently proteolyze them for surrogate peptide identification [47]. However, identification of proteins via surrogate peptides precludes detection of protein variants not known *a priori*. Therefore, it is advantageous to interrogate intact (native) proteins. The single-step immunoaffinity capture provided a seamless interface between the sample and the mass spectrometer: the process was fast (<30 min per batch of 96 samples), multiplexed (96 samples at a time), and cost-effective (similar to ELISAs - the only reagents used were the affinity tips, protein and IRS standards, and the antibodies). The use of polyclonal antibodies assured affinity-retrieval of all variants of the targeted proteins, and the subsequent MALDI-TOF MS analysis presented all variants in a single spectrum.

Detection and quantification of intact proteins provides a full view of the function of the protein (e.g., binding activity, degradation, etc.), and *in-vivo* state of the protein (e.g., presence of protein variants as a result of SNPs and posttranslational modifications). The protein variants question is especially intriguing, ever since “only” ∼25,000 genes have been found to be present in the human genome. Hence, protein diversity play an important role in biological processes, and it is imperative that we engage in its study and delineation. Some protein variants are common, perhaps even ubiquitous. There is already abundant evidence to suggest that some protein variants play distinct biological roles and are critical to the process of health and/or disease. As with the native proteins themselves, there is a great need for reference values for these protein variants that are representative of healthy humans, and that are presented in a manner that can be utilized by all laboratories [48]. We consider this work to represent the first such step toward building a catalog of protein variants concentration ranges and their longitudinal change among the healthy population.

## Supporting Information

Figure S1 Representative mass spectra of the four proteins.
(PPTX)

Figure S2 Representative standard curves for determination of the protein concentrations.
(PPTX)

Figure S3 Concentrations of cysC, RBP, and their variants among the 500 individuals as a function of the state of origin (TN and TX).
(PPTX)

Figure S4 Concentrations of the four proteins and their variants among the 500 individuals as a function of gender.
(PPTX)

Figure S5 Concentrations of cysC, RBP, TTR, and their variants among the 500 individuals as a function of age.
(PPTX)

Figure S6 B2m, RBP, and TTR variants longitudinal concentration profiles for the four individuals over a 27-week period.
(PPTX)
